# Developing Repair Materials for Stress Urinary Incontinence to Withstand Dynamic Distension

**DOI:** 10.1371/journal.pone.0149971

**Published:** 2016-03-16

**Authors:** Christopher J. Hillary, Sabiniano Roman, Anthony J. Bullock, Nicola H Green, Christopher R. Chapple, Sheila MacNeil

**Affiliations:** 1 Kroto research Institute, University of Sheffield, Broad Lane, Sheffield, United Kingdom; 2 Royal Hallamshire Hospital, Glossop Road, Sheffield, United Kingdom; Wake Forest Institute for Regenerative Medicine, UNITED STATES

## Abstract

**Background:**

Polypropylene mesh used as a mid-urethral sling is associated with severe clinical complications in a significant minority of patients. Current *in vitro* mechanical testing shows that polypropylene responds inadequately to mechanical distension and is also poor at supporting cell proliferation.

**Aims and Objectives:**

Our objective therefore is to produce materials with more appropriate mechanical properties for use as a sling material but which can also support cell integration.

**Methods:**

Scaffolds of two polyurethanes (PU), poly-L-lactic acid (PLA) and co-polymers of the two were produced by electrospinning. Mechanical properties of materials were assessed and compared to polypropylene. The interaction of adipose derived stem cells (ADSC) with the scaffolds was also assessed. Uniaxial tensiometry of scaffolds was performed before and after seven days of cyclical distension. Cell penetration (using DAPI and a fluorescent red cell tracker dye), viability (AlamarBlue assay) and total collagen production (Sirius red assay) were measured for ADSC cultured on scaffolds.

**Results:**

Polypropylene was stronger than polyurethanes and PLA. However, polypropylene mesh deformed plastically after 7 days of sustained cyclical distention, while polyurethanes maintained their elasticity. Scaffolds of PU containing PLA were weaker and stiffer than PU or polypropylene but were significantly better than PU scaffolds alone at supporting ADSC.

**Conclusions:**

Therefore, prolonged mechanical distension *in vitro* causes polypropylene to fail. Materials with more appropriate mechanical properties for use as sling materials can be produced using PU. Combining PLA with PU greatly improves interaction of cells with this material.

## Introduction

In the UK, there is a 3.6% lifetime risk for a female patient to undergo surgery for stress urinary incontinence [1]. The most common surgical repair technique involves the use of non-degradable polypropylene (PPL) mesh to support the urethra and counteract sphincter weakness in the treatment of SUI [2]. These repurposed hernia repair meshes became popular due to their success, their ease of use, and the limitations of biological alternatives [3, 4]. Allografts and xenografts have high recurrence rates and tissue encapsulation respectively [5, 6]. Autologous fascia is arguably the most appropriate material for surgical repair but requires longer operating times, can result in donor site morbidity and some patients have insufficient tissue for this to be a viable option.

Use of mesh for the treatment of SUI was popularized by Petros *et al* [7] in 1990, based on the integral theory and the recognition that sphincteric weakness leads to a severe subgroup of SUI, which is not adequately treated with conventional open surgery [8] and was commercialized in 1995. However, in 2013, after nearly two decades of PPL mesh use for the treatment of SUI, the US Food and Drug Administration (FDA) began publishing notifications on the safety of PPL mesh devices due to the increasing awareness of mesh complications. Manufacturers have subsequently withdrawn several mesh implants for the treatment of pelvic organ prolapse (POP), while there is increasing concern over the use of this mesh for the treatment of SUI as complications can take several years to present

Mesh exposure has been reported in over 4% of patients undergoing a trans-vaginal tape (TVT) procedure for SUI [9, 10]. Although the exact mechanism involved in the development of mesh related complications is not completely understood, current literature supports the view that PPL mesh exposure is due to poor tissue integration, host immune attack and excessive fibrosis of the implant [11]. It has been proposed that the biomechanical mismatch between the strong, rigid PPL mesh and the elastic paravaginal tissue, under constant dynamic distension, can lead to PPL becoming plastically deformed [12]. This is supported by *in vivo* data that demonstrates that PPL mesh implanted in a sheep vagina extrudes within 2–3 months but not when implanted abdominally [13], illustrating the site-specific responses to PPL. In the female pelvic floor, any repair material must survive years of dynamic distension. A study using a newly developed device for measuring intra-vaginal pressure in women has elucidated the acute forces that occur during sudden increases in abdominal pressures, such as sneezing, coughing, and laughing [14], which may have been previously under appreciated.

Tissue engineering approaches to develop materials for pelvic floor repair that can lead to long-term success have recently begun to be explored [15–17]. The “ideal” repair material should remain relatively elastic to cope with the forces experienced with routine events such as coughing or sneezing, but become reversibly stronger at higher strain, similar to native healthy fascia [18]. Furthermore, materials should be biocompatible and reflect the properties of the tissues into which it is implanted [19]. Biodegradable materials ideally undergo controlled degradation over a period that permits tissue remodeling (an M2 macrophage response) with fibroblast ingrowth, ECM production, and angiogenesis [20]. Non-degradable materials that result in an acute inflammatory response, persisting to a chronic phase (M1 macrophage response) may be associated with infection and erosion [21], while materials that fail to initiate an M2 response can become encapsulated [22].

Our aim is to design an electrospun sling for SUI, which mimics autologous tissue. As a key step towards this we here explore polyurethanes, which demonstrate greater elasticity and biocompatibility than polypropylene when used in abdominal hernia repair [20]. We also investigate a combination of polyurethanes and electrospun poly-L-lactic acid scaffolds as the latter show good cell attachment and matrix production *in vitro* [15], and these became well integrated on implantation into rats over 7 days [23].

## Materials and Methods

### Polymers

Poly-L-lactic acid ((PLA) Goodfellow, Cambridge, UK)) at 10% (wt/v) was dissolved in dichloromethane (DCM). Polyurethanes (PU) Z1 and Z3 (Biomer technologies, Cheshire, UK) were dissolved in 50:50 dimethylformide:tetrahydrofuran (DMF:THF) at 6% (wt/v) and 70:30 DMF:THF at 10% (wt/v) respectively. PPL mesh (Gynecare^TM^, Johnson & Johnson) was used as supplied.

### Electrospinning

Polymer solutions (20mls total) were loaded into 5ml syringes fitted with blunt tipped 21G needles, placed into a syringe pump (Genie^TM^Plus, Kent Scientific, USA), and delivered at 40μl/min per syringe. Microfibres were created with an accelerating voltage of 17kV DC from a high voltage supply (Genvolt, UK) and collected on an aluminium foil covered earthed mandrel (80mm diameter, 160mm length) rotating at 300rpm, with a needle to collector distance of 17cm at 21°C and ~30% humidity.

**Fig 1:**
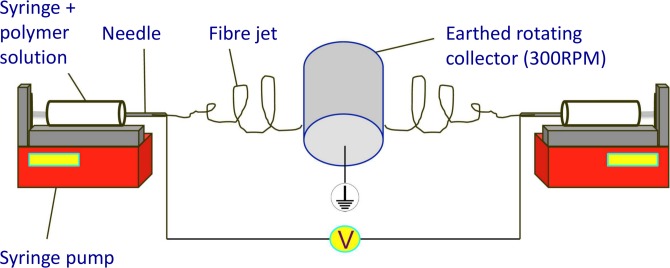
Apparatus for co-electrospinning.

Co-polymer scaffolds of Z1:PLA were formed by simultaneously delivering two individual polymer solutions to the collector from polymer delivery equipment placed either side of the mandrel as depicted in Fig 1. These co-polymers consisted of either 4 syringes of Z1 to 1 syringe of PLA (4:1 Z1 to PLA termed Z1 high (20%) PLA) or 10 syringes of Z1 to 1 syringe of PLA (10:1—Z1 low (9%) PLA). Scaffolds were dried at room temperature for 24 hours prior to storage at -20°C.

### Testing Biomechanical Properties of Scaffolds under Static Conditions and following Dynamic Loading

Samples of all materials were placed in a tensiometer (BOSE Electroforce instruments, Minnesota, USA). Mechanical properties were measured using a ramp test, elongating the material at a rate of 0.1 mm/s or a cyclic test with a rate of 1mm/s up to 25% of displacement from its original length at 50 cycles. Results are standardized by width and thickness of materials (N/mm^2^)

For dynamic loading, samples measuring 3cm x 1cm were placed in a TC-3 load bioreactor (EBERS Medical Technology SL, Zaragoza, Spain) and subjected to cyclic uniaxial distension using 25% elongation, 0.1mm/s rate and 18 cycles per minute over 7 days in Dulbecco’s modified Eagle’s medium (DMEM) at 37°C, 5% CO_2_. Samples were then assessed for mechanical properties as above.

Data was plotted as stress vs strain and the initial linear gradient of each curve was taken as the Young’s modulus (N/mm^2^). Both values were compared to values published for paravaginal tissues of healthy premenopausal patients [24].

### Adipose-Derived Mesenchymal Stem Cell (ADSC) Culture

ADSC were isolated from human subcutaneous fat, donated by patients giving informed consent under a research tissue bank license (number 08/H1308/39) under the Human Tissue Authority. Isolation and culture were performed as previously described from 10 mL of fat tissue [25].

Cells were cultured in DMEM supplemented with 10% (v/v) fetal calf serum (FCS) (Advanced Protein Products, Brierley Hill, UK), 2mM glutamine, 0.625μg/mL amphotericin B, 100IU/mL penicillin and 100μg/mL streptomycin (Gibco Invitrogen, Paisley, UK).

### Sample Preparation and Culture of Cells on Scaffolds

Scaffolds were cut to 1.5 x 1.5cm and sterilized in 70% ethanol for 20 minutes followed by 3 washes in PBS.

500,000 passage 6 ADSC were seeded per scaffold into the centre of steel rings (internal diameter 1cm) placed onto each scaffold, creating a defined area for cell attachment. Rings were removed after 12 hours and samples cultured for 2 weeks at 37°C, 5% CO_2_. DMEM was changed three times per week.

### Assessment of Cell Metabolic Activity on Scaffolds via AlamarBlue™ Assay

The metabolic activity of cells cultured on scaffolds was quantified by AlamarBlue™ (Resazurin; Sigma-Aldrich, Dorset, UK) assay at days 7 and 14. Media was removed and scaffolds were washed three times with PBS. 1mL per well of sterile Resazurin (5mg/ml) was added and cells incubated for one hour. 50μl of each sample was aspirated and the optical density measured at 570nm using a colorimetric plate reader.

### Assessment of Collagen Production on Scaffolds Using Sirius Red Staining

Total collagen production was measured for each scaffold. Following three washes with PBS, 1ml of 0.1% solution of Sirius Red F3B in saturated picric acid was added and samples incubated for 18 hours at room temperature. Samples were washed with PBS until no further stain was eluted. Samples were then weighed and photographed. Stain was eluted using 1ml per well of 0.2M NaOH:MeOH (1:1), and the optical density measured at 490nm using a colorimetric plate reader.

### Examination of Cell Penetration into Scaffolds

Several methods were used to image cell penetration into scaffolds.

DAPI staining of cell nuclei was done post culture of cells on scaffolds. Samples were fixed for 20 minutes in 3.7% formaldehyde and incubated for 40 min in 0.8 ml of 1 ng/ml DAPI (Gibco Invitrogen, Paisley, UK). After three washes in PBS, constructs were imaged with an Axon ImageXpress^TM^ fluorescence microscope (Molecular Devices limited, Union City, CA) at an excitation and emission wavelengths of λ_ex_385 nm/λ_em_461 nm.

Scanning electron microscopy of cells on scaffolds for these same fixed samples was also undertaken. Samples were processed as previously described [25] and gold sputter coated (Edwards sputter coater S150B, Crawley, England). Samples were imaged using a Phillips XL-20 scanning electron microscope (Cambridge, UK).

For imaging of live cells within scaffolds, a fluorescent dye was used to label the cells and second harmonic generation was used to image the scaffolds.

500,000 ADSC were seeded on each of the 5 sterilised scaffolds as previously described and incubated with media (DMEM) changed three times per week. Cell-scaffolds were cultured for 3 weeks, following which, 0.5mls of serum free DMEM with 10μM celltracker™ red CMTPX (Invitrogen, Oregon USA) was added per well and incubated for one hour. Cells were imaged live, using a Zeiss LSM 510 Meta upright laser-scanning confocal microscope (Carl Zeiss MicroImaging, Germany) using a 40x 1.3 NA oil immersion objective attached to a tuneable (700–1060 nm) Chameleon Ti:sapphire multiphoton laser (Coherent, CA, USA) for second harmonic generation (SHG) signal. Red cell tracker signal was created by illuminating constructs at 543nm with 30% transmission and detected between 565nm and 615nm.

For SHG signal, constructs were illuminated at 840nm and signals were detected between 415nm and 426nm. Images (512 x 512), with a pixel dwell time of 6.39 μs were captured at a range of depths by moving the focal plane down from the surface of the scaffold, where there was the greatest number of cells present and without any polymer fibres visible, at 1μm intervals until no further cells were visible and polymer fibres dominated the field of view.

### Statistics

Statistical significance was determined using a two-sample T test with equal variance not assumed.

## Results

### Electrospinning of Scaffolds

**Fig 2:**
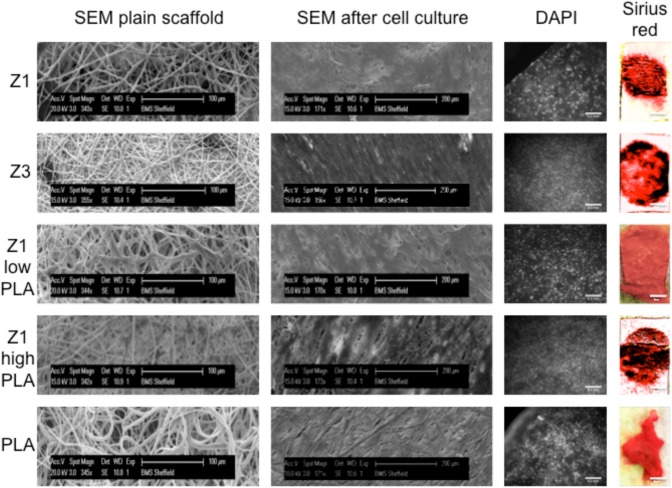
Scanning electron microscopy images of scaffolds before cell seeding and after 2 weeks of culture with ADSC. Cells stained with DAPI and Sirius red.

Scaffolds were either electrospun individually (PLA, Z1 or Z3) or co-electrospun to produce interwoven fibres of Z1 and PLA. All electrospun materials had a microfibrous, microporous structure. PLA had a mean fibre diameter of 2.5μm (shown previously [26]) compared to polyurethane fibres of 1μm. Pore size was 40μm and 20μm respectively. Co-polymers of PLA and Z1 resembled PLA, particularly at the higher PLA content (Fig 2).

### Cell Culture on Scaffolds

Fig 2 shows SEM of ADSC cultured on scaffolds for 2 weeks, demonstrating dense surface matrix coverage. DAPI staining shows cells growing throughout the scaffolds and producing collagen as depicted by staining with Sirius red.

**Fig 3:**
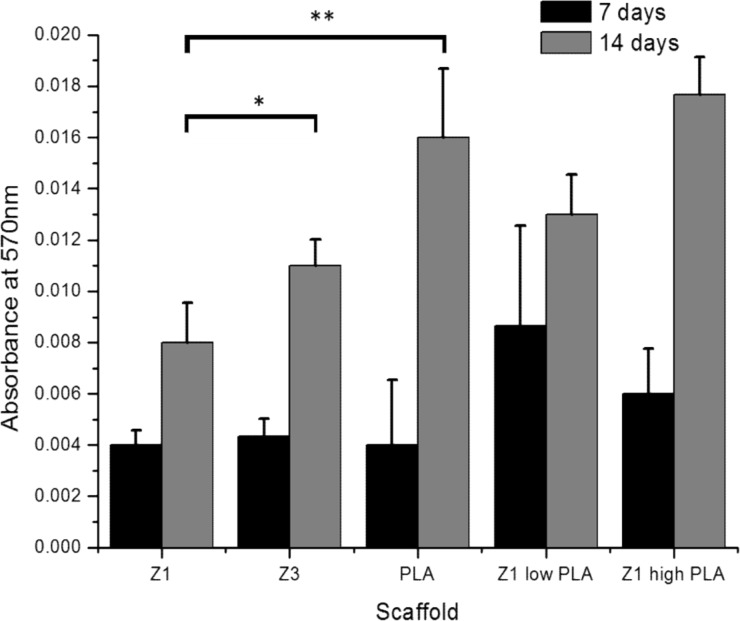
Proliferation of cells on scaffolds assessed by assessing cell metabolic activity with AlamarBlue assay (Absorbance measured at 570nm). (n = 3 ±SEM). *p<0.05 **p<0.01.

A quantitative assessment of these results is shown in Fig 3 and S1 Table. All scaffolds (n = 9) supported an increase in cell viability from day 7 to day 14, however the greatest increase was seen in those scaffolds containing PLA.

**Fig 4:**
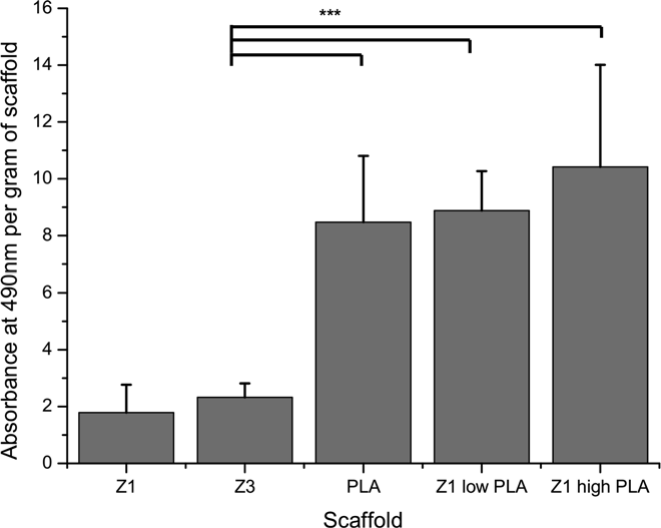
Collagen production of cells cultured on scaffolds. This was measured by Sirius red assay. Absorbance measured at 490nm per gram of scaffold, n = 3±SEM ***p<0.001.

Cells cultured on scaffolds of either PLA or PLA/Z1 showed a significant four-fold increase in total collagen expression compared to that seen on Z1 or Z3 alone after 14 days of culture (n = 9) (Fig 4 and S2 Table).

### Assessment of Mechanical Properties of Scaffolds

**Fig 5:**
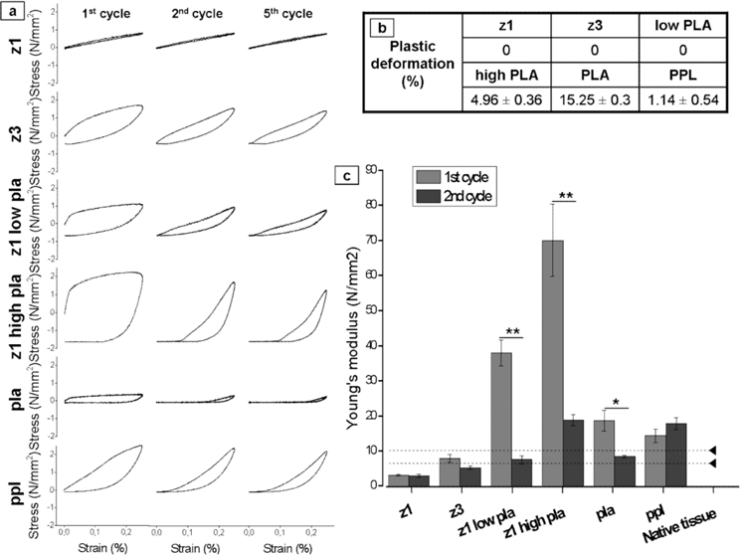
Cyclic uniaxial tensile test of the different materials. (a) Strain-stress plots of the 1^st^, 2^nd^ and 5^th^ cycle. (b) Plastic deformation (%) at the 2^nd^ cycle. (c) Young’s modulus of the 1^st^ and 2^nd^ cycle (n-3±SEM), *p<0.05, **p<0.01.

Fig 5A shows stress-strain curves for the 1^st^, 2^nd^, and 5^th^ cycles of uniaxial tensiometry of dry scaffolds. Percentage deformation (shown in Fig 5B) was calculated as the percentage of change in strain prior to stress at cycle 2. This shows that Z1 was completely elastic, as was Z3, and Z1 with low (9%) PLA co-polymer. By contrast PLA and Z1 with high (20%) PLA underwent significant deformation. PPL showed plastic deformation but to a lesser degree than either PLA or high PLA co-polymers. In addition, all scaffolds containing PLA showed a significant reduction in the Young’s modulus from the 1^st^ to the 2^nd^ cycle (Fig 5C).

**Fig 6:**
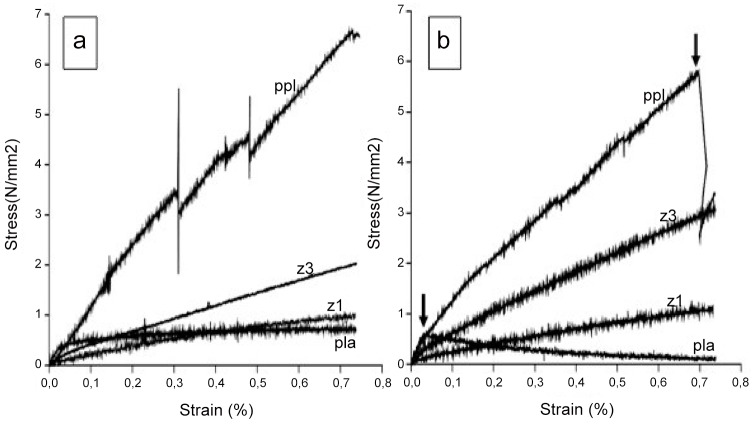
Stress vs strain plots of the 4 different materials. Ramp uniaxial tensile test before (A) and after (B) 7 days under dynamic distention in an EBERS bioreactor.

PPL, PLA, Z1, and Z3 were subsequently assessed before and after 7 days of dynamic distension and stress strain curves are shown in Fig 6. After only 7 days of dynamic distention, PLA became brittle and failed, while PPL increased in stiffness before failing (as indicated by arrows). In contrast Z3 and Z1 both remained elastic.

**Fig 7:**
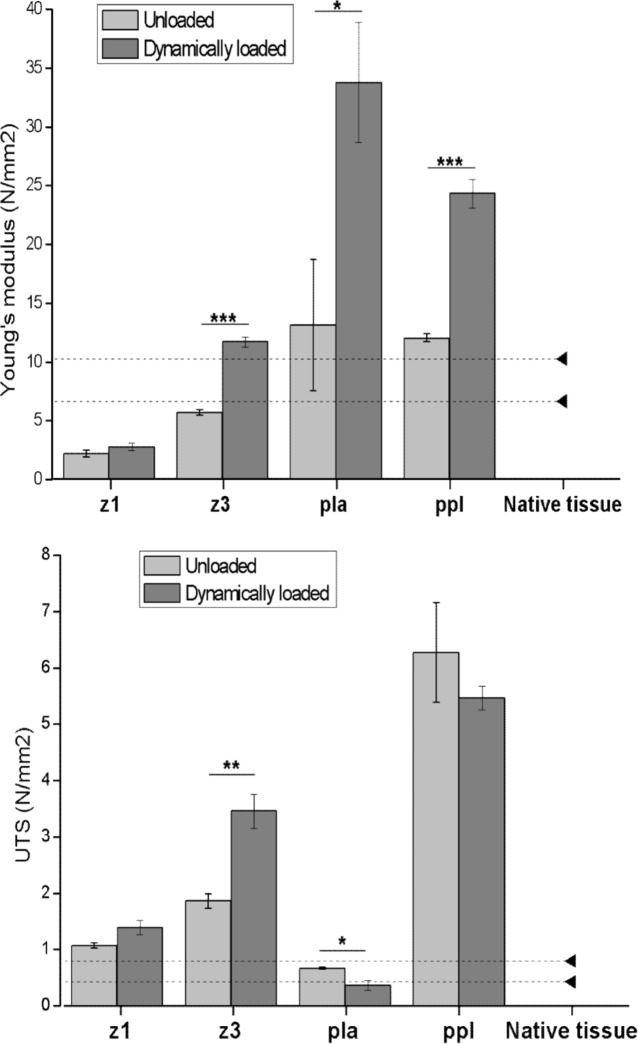
Values for Mechanical properties before and after 7 days of dynamic loading. Young’s Modulus (**top**) and ultimate tensile strength (**bottom**) calculated from stress curves before and after 7 days of uniaxial distension (n-2±SEM), *p<0.05, **p<0.01, ***p<0.001. Dotted lines represent values of healthy paravaginal tissues [24].

Fig 7, S3 Table and S4 Table shows the Young’s modulus and ultimate tensile strength (UTS) for each scaffold in comparison with healthy paravaginal tissues (indicated by the dotted lines) [24]. Only Z1 did not show any changes in Young’s modulus and UTS after 7 days under dynamic loading. Z3 became stiffer and stronger, PLA became stiffer and weaker while PPL became stiffer, its strength unchanged.

### Investigation of Cell Penetration into Scaffolds Using Fluorescence Microscopy and Second Harmonic Generation

**Fig 8:**
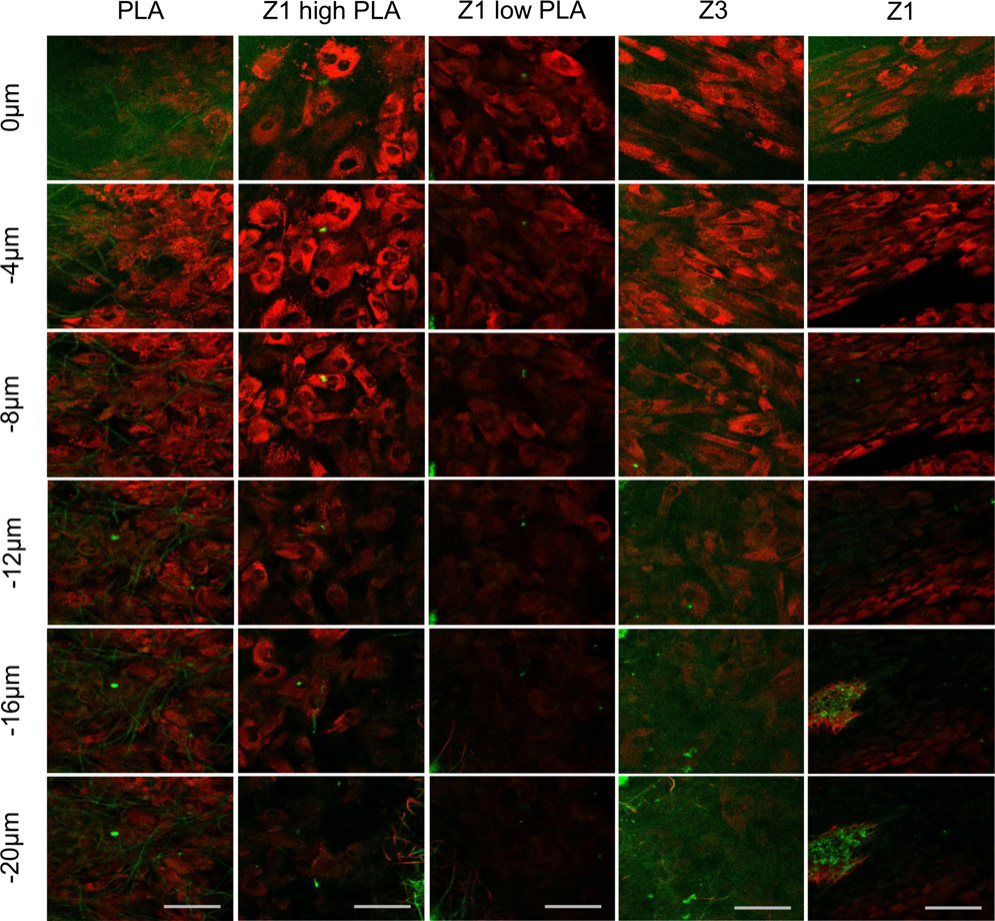
Fluorescence microscopy and scaffold fibre second harmonic generation using confocal microscopy. Cells (**red**) and fibre SHG signal (**green**) for each of the 5 constructs imaged from the scaffold surface (0μm) to 20μm depth. All scale bars equivalent to 50μm.

Unfixed constructs were imaged after 3 weeks of culture at 1μm intervals from the surface (point 0). Red cell-tracker signals were combined with SHG signals for each interval and the results at 4 μm intervals are presented in Fig 8. This demonstrates that cells were present within the PLA fibre pores and were able to penetrate this scaffold to the greatest degree of all constructs tested, followed by the polyurethane Z3 scaffold. Meanwhile, a dense collection of cells was located solely on the surface of polyurethane Z1 scaffolds, without any evidence of cells within polymer fibres. For Z1 high PLA and Z1 low PLA scaffolds, cells were able to integrate within the pores to a lesser degree than PLA scaffolds.

Table 1 summarises the mechanical properties of the scaffolds, including their response to repeated dynamic strain and their ability to support cell proliferation and matrix production.

**Table 1:** Summary of scaffold properties.

SCAFFOLD	ULTIMATE TENSILE STRENGTH	RIGIDITY	RESPONSE TO DISTENSION	CELL PERFORMANCE		
				Cell viability	Collagen production	Cell penetration
Z1	+	+	+	0	0	0
Z3	++	++	++	+	0	+
Z1 low PLA	0	0	N/A	0	++	+
Z1 high PLA	0	0	N/A	+	++	+
PLA	0	0	0	++	++	++
PPL	0	0	0	N/A	N/A	N/A

**Ultimate tensile strength**—**0**(<1N/mm^2^ or >5N/mm^2^), **+**(1-2N/mm^2^), **++**(2-4N/mm^2^). **Rigidity** (approximation to healthy tissue)– **0**(>200%), **+**(50%-100%), **++**(<50%). **Response to distension** (Young’s Modulus approximation to healthy tissue) - **0**(>200%), **+**(50–100%), **++**(<50%). **Cell viability** (from 7 to 14 days)– **0**(<100%), **+**(100%-200%), **++**(>200%). **Collagen production** (% increase from Z1)– **0**(<100%), **+**(100–200%), **++**(>200%). **Cell penetration** (ability of cells to penetrate scaffold pores)– **0**(no penetration), **+**(cells and fibres present), **++**(cells present within fibre pores).

## Discussion

Our aim was to design a biocompatible repair material sling for the treatment of stress urinary incontinence, in light of the complications associated with polypropylene mesh. We investigated the use of electrospun polyurethane scaffolds to replicate the mechanical properties of healthy fascia and also assessed cell attachment and matrix production, as *in vivo* post implantation this is expected to occur. Any materials for load-bearing must have adequate mechanical properties to fulfill a supportive role of the weakened tissue in addition to being biocompatible.

The key finding of this study is that subjecting materials *in vitro* to dynamic strain reveals significant changes in their mechanical properties after only 7 days. PPL and PLA both failed mechanically, while polyurethanes Z1 and Z3 coped well with dynamic strain. However, PLA is superior at supporting cellular interactions and new matrix production. We suggest that this dynamic assessment is crucial in the development of materials for use in the pelvic floor.

Human ADSC were used as a cell source for this study for investigating the interaction of materials with cells, as these cells are more proliferative and better defined than fibroblasts [27] and *in vivo* may well be recruited to implanted biomaterials.

The implantation of a weak material could lead to recurrence of SUI, while a strong but inelastic material, such as PPL will provide mechanical support but is ultimately incompatible with the pelvic floor environment, which could lead to fibrosis and chronic inflammation.

Table 1 summarises the key properties of the investigated materials, showing polyurethane Z3, which is slowly degradable (over 5–10 years) has mechanical characteristics similar to native fascia. By interweaving fibres of Z1 with PLA, we significantly improved the interaction of the scaffolds with cells, despite a reduction in material strength and the ability of cells to penetrate the scaffolds.

In developing materials for the pelvic floor, it was not appreciated until recently that there are site-specific differences seen with “soft” tissues. Evidence from studies using sheep models show that while PPL performs well in the abdominal wall, it extrudes through pelvic floor tissues within months of implantation into the vagina [13]. It has also been demonstrated recently that although polypropylene mesh is strong, it is unsuited to dynamic distension with irreversible deformation during cyclical loading [12].

There is no simple correlation between the strength of implants and clinical success [18]. Some non-degradable biomaterials lead to sustained inflammation [11] and are associated with complications years after their use for POP or SUI. Thus, biomaterials for use in the pelvic floor need to be both mechanically suitable and not provoke sustained inflammation.

Accordingly we evaluated polyurethanes as potential graft materials. These are popular in vascular and bone tissue engineering due to their elasticity and biocompatibility. Bergmeister *et al* [28] demonstrated endothelial cell proliferation and 100% graft patency in cylindrical PU grafts one-year post implantation. Takanari *et al* [29] showed greater elasticity and anti-inflammatory properties, of polyurethanes compared to PPL and other potential repair materials for hernia repair Hence we investigated the mechanical properties of electrospun scaffolds of polyurethanes, of PLA and PPL and of combinations of PU and PLA and compared these values to those reported in the literature for native healthy fascia. We also assessed cell interactions with these materials and demonstrated that cells are able to penetrate PLA scaffolds and to a lesser degree, polyurethane Z3.

In conclusion, the surgical repair of SUI and particularly the implantation of synthetic materials into the pelvic floor environment raises particular challenges that need to be considered in order to design appropriate new materials, which will provide a sustained and successful repair without the complication observed with PPL. We demonstrate that it is possible to develop a composite PU/PLA material, which out-performs PPL both in response to dynamic distension and in its ability to interact well with cells *in vitro*. We suggest a combination of these FDA approved materials could be more suitable than PPL for successful implantation and long-term survival for the management of both SUI and POP based on this *in vitro* work.

## Supporting Information

S1 Table Raw data for cell metabolic activity testing. (XLSX)

S2 Table Raw data for total collagen production. (XLSX)

S3 Table Raw data for tensile and cyclical uniaxial mechanical testing. (XLS)

S4 Table Raw data for dynamically distended materials. (XLS)
